# Serum Vitamin Profiles in Pediatric Eczema, Atopic Dermatitis, and Urticaria

**DOI:** 10.3390/nu18111754

**Published:** 2026-05-29

**Authors:** Gaolei Zhang, Mengting Su, Xiao Liu, Xiaoyan Liu, Jianyou Chen, Sheng Zhang, Yuhan Wang, Guimin Huang, Tao Li

**Affiliations:** 1 Department of Dermatology and Venereology, Capital Center for Children’s Health, Capital Medical University, Capital Institute of Pediatrics, Beijing 100020, China; pkuzhgl@163.com (G.Z.); sumengting2019@163.com (M.S.); liuxiao_0204@163.com (X.L.);; 2 Child Health Big Data Research Center, Capital Center for Children’s Health, Capital Medical University, Capital Institute of Pediatrics, Beijing 100020, China

**Keywords:** children, inflammatory skin diseases, vitamins, eczema, atopic dermatitis, urticaria

## Abstract

**Background**: Eczema, atopic dermatitis, and urticaria are common pediatric inflammatory skin diseases, but serum vitamin profiles across these diseases remain poorly characterized. **Objectives**: To compare demographic characteristics, serum vitamin levels, and vitamin insufficiency rates among children with these diseases, and to identify independent factors associated with disease presence. **Methods**: This retrospective study included 504 children: 43 with eczema, 43 with atopic dermatitis, 40 with urticaria, and 378 healthy controls. Serum levels of nine vitamins were measured by electrochemical assays. Univariable and multivariable logistic regression analyses were used to identify associated factors with false discovery rate correction. Propensity score matching based on age and sex was additionally performed for each disease-control comparison, followed by matched regression analyses. An exploratory nomogram was developed and evaluated. **Results**: The mean age of the cohort was 6.26 years, and 50.2% were male. Vitamin B9 insufficiency was the most prominent abnormality, occurring more frequently in the overall disease group than in controls (17.5% vs. 0.3%, *p* < 0.001). Vitamin D insufficiency appeared more frequently in the urticaria group than in controls (42.5% vs. 28.3%, *p* = 0.062). In multivariable analyses after PSM, vitamin B9 insufficiency and lower vitamin B6 levels remained independently associated with all three diseases. **Conclusions**: Pediatric inflammatory skin diseases exhibited distinct vitamin profiles relative to healthy controls, with vitamin B9 insufficiency emerging as a common feature across eczema, atopic dermatitis, and urticaria.

## 1. Introduction

Eczema, atopic dermatitis, and urticaria are common parts of the pediatric disease burden worldwide [1,2,3]. These diseases present several clinical features, including pruritus, recurrent episodes, and a substantial impact on physical health, psychosocial well-being, and quality of life, making comparative analysis clinically relevant [1,3,4,5]. Although vitamins have received increasing attention in research on skin diseases, most previous studies have focused on single nutrients or individual diseases at a time, and cross-disease comparisons of multiple vitamins in pediatric populations remain limited [6,7,8].

Accumulating evidence suggests that micronutrients play important roles in the onset, severity, and course of inflammatory skin diseases [6,7,8,9]. Childhood is a nutritionally demanding period of life because of children’s distinct physiological characteristics. Among micronutrients, vitamins are involved in immune function, redox balance, and skin barrier maintenance [7,8,10,11]. Vitamin insufficiency may result from chronic illness, dietary restriction, altered intake, or disease-related metabolic changes, and may in turn aggravate clinical manifestations [10,11,12]. Vitamins D and C have been studied most extensively in skin disease. Vitamin D is important for cutaneous immunity, whereas vitamin C supports collagen synthesis and helps protect the skin from oxidative damage [10,11,13,14].

By comparison, the involvement of B vitamins in pediatric inflammatory skin diseases remains insufficiently studied [6,11]. Vitamin B6 participates in more than 140 enzymatic reactions related to lymphocyte proliferation, antibody production, and cytokine synthesis, and its deficiency may impair both cellular and humoral immunity. Vitamin B2 serves as an essential cofactor for enzymes that maintain redox homeostasis and regulate innate immune responses [11]. Vitamin B9 is essential for DNA synthesis and repair and for the regulation of gene expression through methylation, and its deficiency may therefore contribute to immune dysfunction [15,16]. In a cohort of 582 Puerto Rican children, vitamin B9 insufficiency was associated with more severe allergic manifestations and a higher risk of severe asthma exacerbations, particularly when accompanied by vitamin D insufficiency [16].

However, the evidence published on the associations of eczema, atopic dermatitis, and urticaria with vitamin A, B1, B2, B6, B9, B12, C, D, and E remains limited. In view of this, our study was designed to compare demographic characteristics, serum levels of these vitamins, and vitamin insufficiency rates among children with eczema, atopic dermatitis, urticaria, and healthy controls, and to identify independent factors associated with disease presence. By clarifying both shared vitamin abnormalities and those specific to each disease, this study aimed to improve understanding of the nutritional features of pediatric inflammatory skin diseases and to provide an exploratory clinical basis for future prospective studies on individualized prevention and management.

## 2. Materials and Methods

### 2.1. Study Design

This single-center retrospective study was conducted from July 2018 to June 2025 at the Department of Dermatology, Capital Center for Children’s Health, Capital Medical University. The Institutional Review Board reviewed the study and waived the requirement for informed consent, given the deidentified data.

### 2.2. Study Population

The overall disease group comprised children aged 4 months to 16 years diagnosed with eczema, atopic dermatitis, or urticaria. All patients included had complete electronic medical records and underwent standardized serum vitamin testing within 4 weeks after diagnosis. Diagnoses were independently confirmed by three dermatologists according to the Williams Criteria and the International Urticaria Guideline [17,18]. Eczema was defined as eczematous lesions that did not meet the diagnostic criteria for atopic dermatitis. Exclusion criteria included chronic systemic diseases affecting nutritional metabolism, systemic immunosuppressive therapy within 3 months before testing, continuous vitamin supplementation for more than 3 months before testing, incomplete medical records or missing key variables. During the same period, healthy children attending routine health examinations at our hospital who had completed serum vitamin testing and met the same exclusion criteria were included as controls. Because the number of healthy controls was substantially larger than that of the disease cohort, a 1:3 case-to-control ratio was used to improve statistical power and increase the precision of effect estimates; accordingly, 378 controls were randomly selected for the analysis. In total, 504 participants were included: 126 in the overall disease group (eczema, *n* = 43; atopic dermatitis, *n* = 43; urticaria, *n* = 40) and 378 healthy controls.

### 2.3. Data Collection and Vitamin Assessment

Fasting peripheral venous blood (2 mL) was collected in the morning from participants. Serum was separated and used for the analysis. Serum concentrations of nine vitamins were determined by electrochemical stripping voltammetry using an LK3000VI Vitamin Analyzer (Tianjin LANBIAO Electronic Technology Co., Ltd., Tianjin, China). Instrument quality control was performed before each assay. The specific molecular forms measured were: retinol (vitamin A), thiamine (vitamin B1), riboflavin (vitamin B2), pyridoxal-5′-phosphate (vitamin B6), folic acid (vitamin B9), cyanocobalamin (vitamin B12), ascorbic acid (vitamin C), 25-hydroxyvitamin D (vitamin D), and α-tocopherol (vitamin E). The coefficients of variation were below 10% for all vitamins. Vitamin status was classified as deficient, insufficient, or sufficient according to national pediatric guidelines, with the corresponding cut-off values summarized in Supplementary Table S1 [19,20]. For analysis, deficiency and insufficiency were combined as vitamin insufficiency, and the proportion of vitamin insufficiency was calculated for each group.

Data were extracted independently by two trained research assistants from the electronic outpatient medical record system using a standardized form, and discrepancies were adjudicated by the attending clinician. Collected variables included age, gender, primary diagnosis, date of diagnosis, and serum vitamin measurements.

### 2.4. Statistical Analysis

Statistical analyses were performed using R version 4.4.1 and IBM SPSS Statistics 27. Continuous variables were presented as mean ± standard deviation (SD), and categorical variables as *n* (%). Group comparisons for continuous variables were performed using one-way analysis of variance or the Kruskal–Wallis test, and pairwise comparisons were conducted using the independent-samples *t* test or Mann–Whitney U test. Categorical variables were compared using the chi-square test or Fisher’s exact test. To assess the associations of demographic and vitamin-related factors with diagnostic group, separate binary logistic regression analyses were conducted for each pairwise comparison, including a vitamin-level model with age, gender, and serum vitamin levels and a vitamin-insufficiency model with age, gender, and vitamin insufficiency status. In each model set, variables with *p* < 0.20 in univariable analyses were included in the multivariable model. Firth’s penalized logistic regression was applied in the presence of sparse data or complete/quasi-complete separation. Odds ratios (ORs) with 95% confidence intervals (CIs) were reported. Given the large number of comparisons, the Benjamini–Hochberg false discovery rate (FDR) method was used. Nominal and FDR-adjusted *p* values were both evaluated.

Additionally, propensity score matching (PSM) was performed as a supplementary analysis for each pairwise disease-control comparison using age and gender, with 1:1 nearest-neighbor matching. Covariate balance after matching was assessed using standardized mean differences. In the matched samples, serum vitamin levels were further evaluated using univariable and multivariable conditional logistic regression stratified by matched pair, whereas vitamin insufficiency indicators were primarily analyzed using conditional logistic regression, with exact McNemar tests or Firth’s penalized logistic regression additionally applied when sparse data or separation was present.

An exploratory nomogram was developed based on the independent predictors identified in the multivariable vitamin-level model for the overall disease group versus healthy controls. Internal validation was performed using 1000 bootstrap resamples. Model performance was assessed using the area under the receiver operating characteristic curve (AUC), calibration plots, and decision curve analysis. A two-sided *p* < 0.05 was considered statistically significant.

## 3. Results

### 3.1. Demographic Characteristics of the Cohort

A total of 504 participants were enrolled, including 43 with eczema, 43 with atopic dermatitis, 40 with urticaria, and 378 healthy controls. The overall cohort had a mean age of 6.26 ± 3.97 years and included 50.2% males; corresponding values for the eczema, atopic dermatitis, urticaria, and control groups were 4.14 ± 3.50 and 53.5%, 4.98 ± 3.48 and 48.8%, 5.08 ± 3.26 and 55.0%, and 6.77 ± 4.00 and 49.7%, respectively. The mean age was lower in the overall disease group than in the control group (4.73 ± 3.45 vs. 6.77 ± 4.00 years, *p* < 0.001), with the eczema group having the lowest mean age among the disease groups, whereas gender distribution was comparable across groups (Table 1 and Table 2 and Supplementary Table S2). After propensity score matching (PSM), the matched cohorts of each disease group versus controls showed balanced age and gender distributions (standardized mean differences < 0.1; Supplementary Table S5).

### 3.2. Patterns of Serum Vitamin Levels and Vitamin Insufficiency Rates

Compared with healthy controls, the overall disease group had significantly lower serum vitamin B6 and B9 levels (both *p* < 0.001) (Table 1 and Table 2 and Supplementary Table S2). Across individual disease groups, serum vitamin B6 and B9 levels were significantly lower in the eczema, atopic dermatitis, and urticaria groups than in healthy controls (all *p* < 0.01). In comparisons among the three disease groups, the atopic dermatitis group had lower vitamin B6 levels than the eczema group (*p* = 0.044); however, this difference did not remain significant after FDR correction (*p* = 0.175).

Vitamin insufficiency analysis showed that vitamin B9 insufficiency was the most prominent abnormality in the overall disease group, occurring in 22 of 126 children (17.46%) compared with 1 of 378 healthy controls (0.26%) (*p* < 0.001). The vitamin B9 insufficiency rate was 8/43 (18.60%) in eczema, 7/43 (16.28%) in atopic dermatitis, and 7/40 (17.50%) in urticaria, all significantly higher than that in healthy controls (all *p* < 0.001), with no significant differences among the three disease groups. Vitamin B2 insufficiency occurred in 3/40 children with urticaria (7.50%), which was significantly higher than in healthy controls (0/378, 0.00%; *p* = 0.001). Vitamin D insufficiency was also more frequent in the overall disease group than in healthy controls (47/126 [37.30%] vs. 107/378 [28.31%], *p* = 0.039); however, this association did not remain significant after FDR correction (*p* = 0.165). By contrast, vitamin A insufficiency was highly prevalent in the overall cohort (441/504, 87.50%) but did not differ significantly between patients and healthy controls.

### 3.3. Univariable and Multivariable Logistic Regression Analyses of Factors Associated with Disease Presence

In univariable logistic regression analyses, age, serum levels of vitamin B6 and B9 were significantly associated with overall disease presence (age: OR = 0.86, 95% CI: 0.81–0.91, *p* < 0.001; vitamin B6: OR = 0.93, 95% CI: 0.91–0.95, *p* < 0.001; vitamin B9: OR = 0.88, 95% CI: 0.84–0.92, *p* < 0.001; Table 3 and Supplementary Table S3). Pairwise analyses of the disease groups further identified significant differences between eczema and atopic dermatitis in vitamin B6 levels (OR = 1.04, 95% CI: 1.00–1.09, *p* = 0.049); however, this association did not remain significant after FDR correction (*p* = 0.183). In age- and gender-matched univariable analyses, lower serum vitamin B6 levels remained significantly associated with eczema (OR = 0.94, 95% CI: 0.90–0.99; *p* = 0.018), atopic dermatitis (OR = 0.87, 95% CI: 0.80–0.95; *p* = 0.001), and urticaria (OR = 0.93, 95% CI: 0.88–0.98; *p* = 0.012) versus matched controls. Lower serum vitamin B9 levels were also associated with eczema (OR = 0.93, 95% CI: 0.86–1.00; *p* = 0.047) and atopic dermatitis (OR = 0.91, 95% CI: 0.83–0.99; *p* = 0.029), but not with urticaria (Supplementary Table S6).

In univariable analyses of vitamin insufficiency indicators, vitamin B9 insufficiency showed the strongest association with overall disease presence, with an OR of 76.1 (95% CI: 10.1–572.6; *p* < 0.001). Vitamin D insufficiency was also associated with overall disease presence (OR = 1.57, 95% CI: 1.02–2.40, *p* = 0.039), but this finding did not remain significant after FDR correction (*p* = 0.135). Vitamin B2 insufficiency was specifically associated with urticaria (OR = 70.65, 95% CI: 6.67–9584.50, *p* < 0.001). In matched univariable analyses of vitamin insufficiency, vitamin B9 insufficiency remained significantly associated with eczema (OR = 20.83, 95% CI: 2.45–2727.83; *p* = 0.002), atopic dermatitis (OR = 15.49, 95% CI: 1.73–2045.94; *p* = 0.010), and urticaria (OR = 18.13, 95% CI: 2.08–2385.59; *p* = 0.005). By contrast, vitamin B2 insufficiency in urticaria was not significant in the matched univariable analyses.

**Figure 1:**
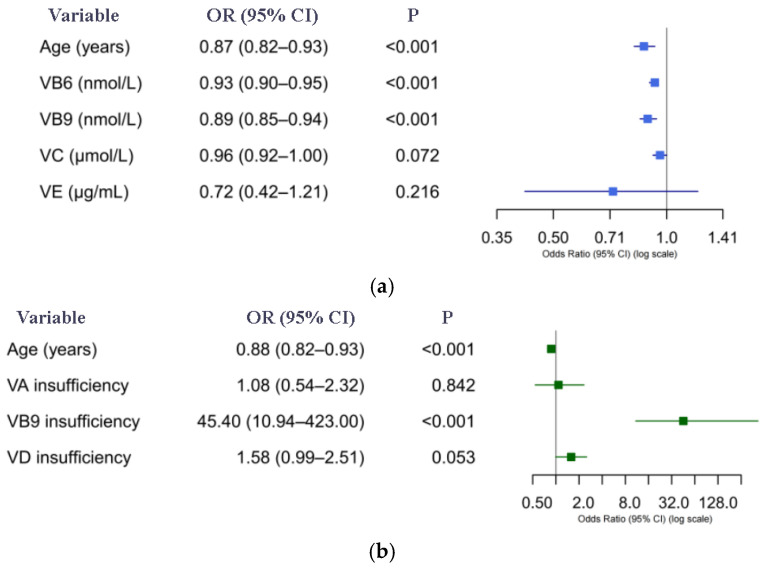
Forest plot of multivariable logistic regression comparing the overall disease group with the control group: (**a**) The continuous vitamin model; (**b**) The vitamin insufficiency model.

In multivariable vitamin-level models, age (OR = 0.87, 95% CI: 0.82–0.93; *p* < 0.001), serum levels of vitamin B6 (OR = 0.93, 95% CI: 0.90–0.95; *p* < 0.001) and vitamin B9 (OR = 0.89, 95% CI: 0.85–0.94; *p* < 0.001) remained independently associated with overall disease presence (Table 4 and Supplementary Table S4; Figure 1a). Pairwise comparisons further showed that, relative to healthy controls, eczema, atopic dermatitis, urticaria were each independently associated with serum vitamin B6 and B9 levels (eczema: vitamin B6, OR = 0.95, 95% CI: 0.91–0.99, *p* = 0.020, and vitamin B9, OR = 0.89, 95% CI: 0.82–0.96, *p* = 0.002; atopic dermatitis: vitamin B6, OR = 0.88, 95% CI: 0.84–0.92, *p* < 0.001, and vitamin B9, OR = 0.86, 95% CI: 0.79–0.93, *p* < 0.001; urticaria: vitamin B6, OR = 0.94, 95% CI: 0.90–0.98, *p* = 0.002, and vitamin B9, OR = 0.87, 95% CI: 0.80–0.95, *p* = 0.001). Comparison between eczema and atopic dermatitis further identified serum vitamin B6 level as a significant distinguishing factor (OR = 1.06, 95% CI: 1.01–1.11, *p* = 0.029); however, this association did not remain significant after FDR correction (*p* = 0.068). These associations were partly confirmed in the matched multivariable analyses. After matching on age and gender, lower serum vitamin B6 levels remained independently associated with eczema (OR = 0.95, 95% CI: 0.90–1.00; *p* = 0.039), atopic dermatitis (OR = 0.85, 95% CI: 0.76–0.95; *p* = 0.004), and urticaria (OR = 0.94, 95% CI: 0.88–0.99; *p* = 0.022) versus matched controls. Lower serum vitamin B9 levels also remained independently associated with atopic dermatitis (OR = 0.79, 95% CI: 0.63–0.98; *p* = 0.031), while the association with eczema was attenuated after matching (OR = 0.94, 95% CI: 0.87–1.01; *p* = 0.112). In urticaria, vitamin B9 was not retained in the matched multivariable model (Supplementary Table S7).

In multivariable vitamin-insufficiency models, vitamin B9 insufficiency remained independently associated with eczema (OR = 28.73, 95% CI: 6.00–279.50; *p* < 0.001), atopic dermatitis (OR = 37.06, 95% CI: 7.20–369.60; *p* < 0.001), and urticaria (OR = 60.76, 95% CI: 11.85–626.70; *p* < 0.001) (Figure 1b). Vitamin D insufficiency was independently associated with urticaria (OR = 2.20, 95% CI: 1.07–4.47; *p* = 0.032), but this association did not remain significant after FDR correction (*p* = 0.061). The matched multivariable analyses yielded consistent results for vitamin B9 insufficiency, which remained independently associated with eczema (OR = 18.50, 95% CI: 2.13–2434.59; *p* = 0.004), atopic dermatitis (OR = 14.50, 95% CI: 1.60–1920.75; *p* = 0.013), and urticaria (OR = 23.35, 95% CI: 2.60–3094.57; *p* = 0.002) versus matched controls. In contrast, vitamin D insufficiency in urticaria was not independently significant after matching.

### 3.4. Development and Validation of an Exploratory Risk Prediction Nomogram

**Figure 2:**
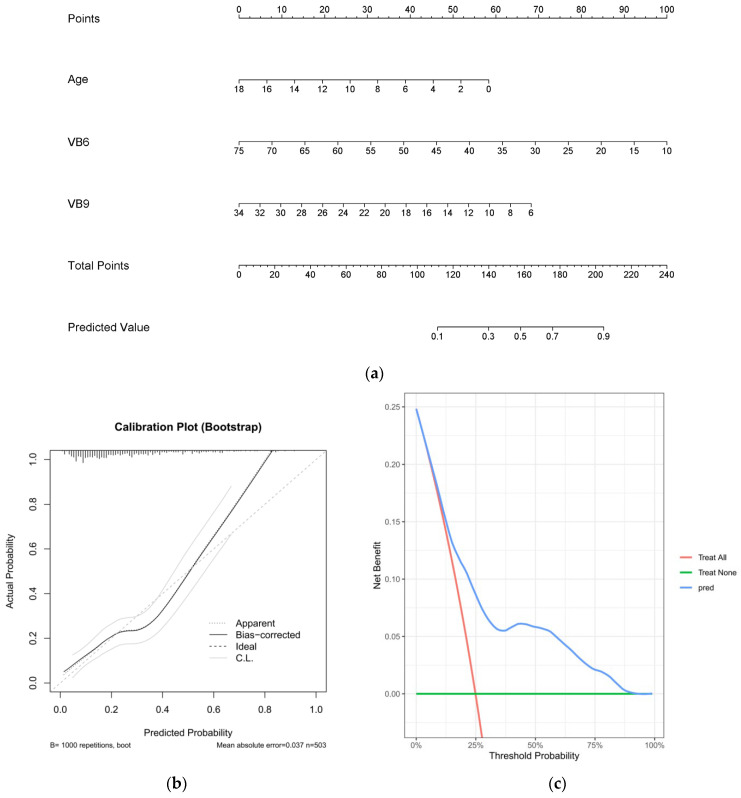
Development and validation of an exploratory nomogram for predicting the probability of overall disease: (**a**) Nomogram; (**b**) Calibration plot; (**c**) Decision curve analysis.

A nomogram was developed as an exploratory tool to estimate the probability of overall disease presence based on three independent predictors identified in the multivariable vitamin-level logistic regression model: age, vitamin B6 and B9 levels (Figure 2a). In the nomogram, younger age and lower levels of vitamins B6 and B9 were assigned to higher point values, corresponding to a higher predicted probability of overall disease. The model yielded an AUC of 0.753 (95% CI: 0.702–0.804). The calibration plot showed good agreement between predicted and observed probabilities, with a mean absolute error of 0.037 (Figure 2b). Decision curve analysis suggested a positive net benefit across threshold probabilities of 10% to 70% (Figure 2c). Although the model performed well in internal validation, it has not yet been externally validated and should therefore be regarded as preliminary.

## 4. Discussion

The broader interaction between nutritional status, inflammation, and oxidative stress has been increasingly recognized in chronic inflammatory disorders [21]. Vitamins are closely involved in skin barrier maintenance, redox regulation, and immune homeostasis [14,22,23,24,25]. Although vitamin D has been linked to atopic dermatitis and urticaria, and vitamins C, E, and B vitamins have also been reported to be associated with allergic diseases [7,14,22,24,26,27,28,29,30], comparative evaluation of serum vitamin profiles across pediatric eczema, atopic dermatitis, and urticaria remains limited. In this study, vitamin B9 insufficiency showed the most consistent association with overall disease presence, whereas lower serum vitamin B6 levels were also associated with all three disease groups. Several other vitamin-related associations were attenuated after FDR correction. These findings suggest that vitamin-related alterations may be relevant to the heterogeneity of pediatric inflammatory skin diseases, although the biological basis of these associations remains unclear.

Vitamin B9 insufficiency was the most prominent shared abnormality across the disease groups. Vitamin B9 insufficiency was significantly associated with eczema, atopic dermatitis, and urticaria in multivariable analyses, and this remained the most robust finding after propensity score matching. Previous studies on vitamin B9 in these three conditions are limited. Therefore, our findings may add to the current evidence on vitamin B9 insufficiency across these conditions. As a key cofactor in one-carbon metabolism, vitamin B9 is involved in nucleotide synthesis, DNA methylation, epithelial renewal, and immune cell proliferation and differentiation [22,31]. As an exploratory finding, the underlying mechanisms of these associations warrant further investigation. However, the large effect estimates observed for vitamin B9 insufficiency should be interpreted cautiously, as its exceptional rarity in healthy controls resulted in wide confidence intervals and limited precision despite the use of Firth’s penalized logistic regression.

Vitamin D also warrants consideration. In the present study, vitamin D insufficiency showed a nominal association with urticaria in multivariable analyses and with the overall disease group in univariable analyses, but these associations did not remain statistically significant after FDR correction. Previous studies have linked vitamin D deficiency to a range of autoimmune and allergic disorders, including atopic dermatitis [32,33]. Similarly, studies in urticaria have shown that compared with healthy individuals, patients with chronic spontaneous urticaria are more likely to have vitamin D deficiency, and that lower vitamin D levels are associated with greater disease activity and poorer clinical outcomes [30,34,35]. In addition, vitamin D may influence the pathogenesis of urticaria by suppressing IgE-mediated mast cell activation, enhancing regulatory T-cell function, and inhibiting Th1/Th17-associated inflammatory responses [31,33,35]. Taken together, our data provided only exploratory support for a possible role of vitamin D insufficiency, particularly in urticaria.

Vitamin B6 emerged as another factor potentially relevant to disease presence. In the multivariable vitamin-level model, lower vitamin B6 levels were independently associated with higher odds of pediatric inflammatory skin diseases. Notably, this association was also reproduced in the propensity score-matched analyses. Although vitamin B6 also showed nominal differences between atopic dermatitis and eczema in the multivariable analyses, these associations did not remain significant after FDR correction. Vitamin B6 is an essential cofactor in amino acid metabolism, neurotransmitter synthesis, and immune regulation, and its active form, pyridoxal 5′-phosphate, is involved in multiple immune-related processes [26,27]. Low vitamin B6 status has also been associated with inflammation and altered oxidative stress responses [26,36]. A recent study reported an association between vitamin B6 status and eczema in children and adolescents, whereas evidence in atopic dermatitis is largely indirect and data in urticaria remain sparse [7,37]. Despite statistically significant between-group differences, vitamin B6 levels remained within the reference range across all groups. Large-scale, multicenter studies are needed to clarify the clinical significance of these differences.

In the present study, vitamin A insufficiency and deficiency were highly prevalent across all groups (86.24–95.35% and 25.00–38.89%, respectively), without significant between-group differences. This pattern suggests that suboptimal vitamin A status may reflect a background nutritional issue in the study population rather than a feature specific to inflammatory skin diseases. Previous studies in Chinese children reported rates of vitamin A insufficiency (24.29–59.5%) and deficiency (5.16–15.7%) [9,38,39,40]. Consistent with global evidence that vitamin A deficiency remains common in some pediatric populations [41], our findings underscore the continuing public health relevance of inadequate vitamin A status. Although no disease-specific association was identified in this study, vitamin A is known to be involved in skin barrier function and immune regulation. Previous studies have suggested that vitamin A deficiency may impair barrier repair and host defense, disrupt immune regulation, and promote inflammation, thereby increasing susceptibility to infection and potentially exacerbating inflammatory skin diseases [42,43,44,45]. In addition, altered vitamin A metabolism in atopic dermatitis suggests a role for local retinoid dysregulation in epidermal hyperproliferation and cutaneous inflammation [46]. Nevertheless, whether this high prevalence reflects population-level nutritional patterns, assay-related differences, or age-specific characteristics remains unclear.

Additional vitamin-related findings also deserve comment. In the univariate analysis, vitamin B2 insufficiency was significantly more common in the urticaria group than in controls (*p* < 0.001). However, this association was not confirmed in the matched analyses. Given the established role of vitamin B2 in flavin-dependent energy metabolism, redox homeostasis, and antioxidant defense, this finding may warrant further investigation in relation to metabolic and oxidative pathways in urticaria [23,30,47]. In multivariable analyses, vitamin C levels showed a nominal association with eczema relative to healthy controls, but this did not remain significant after FDR correction. Vitamin C is a major water-soluble antioxidant involved in reactive oxygen species scavenging, epithelial support, and skin health, with recognized dermatological and pharmaceutical relevance [48]. Low vitamin C status has been reported to aggravate eczema and chronic inflammation, whereas adequate dietary intake may help support antioxidant defense and cutaneous homeostasis [7,48,49]. Nevertheless, these findings were based on a low frequency of vitamin insufficiency in all groups; they should be interpreted cautiously.

All multivariable analyses were adjusted for age and gender, and additional propensity score-matched analyses were performed to further minimize confounding by these baseline factors. Although age was independently associated in some models with the disease groups—particularly the eczema group—having a lower mean age than the control group [50,51], some age-related differences between groups were attenuated after FDR correction. Nevertheless, the associations between disease presence and vitamin profiles, particularly for vitamin B9 insufficiency and lower vitamin B6 levels, remained independently significant after adjustment [22,25,27]. The persistence of these associations in the matched analyses further suggests that the observed alterations in vitamin status are unlikely to be explained solely by age imbalance and may instead be related to disease presence. Given the retrospective single-center design and the fact that diagnoses were based on the index visit, potential diagnostic overlap or evolution between eczema and atopic dermatitis could not be assessed. Moreover, several potentially important confounders, such as diet, sunlight exposure, nutritional status, socioeconomic background, and other clinical factors, were unavailable. Accordingly, the findings warrant confirmation in studies incorporating longitudinal follow-up, age-stratified designs, and more comprehensive clinical data.

## 5. Conclusions

In multivariable analyses, vitamin B9 insufficiency was independently associated with eczema, atopic dermatitis, and urticaria. Larger prospective studies are needed to validate these associations, and interventional controlled studies will be required to assess their potential clinical significance.

## Figures and Tables

**Table 1:** Demographic characteristics, serum vitamin levels, and vitamin insufficiency rates among the study groups.

Variables	Total (*n* = 504)	Control (*n* = 378)	Disease (*n* = 126)	Eczema (*n* = 43)	AD (*n* = 43)	Urticaria (*n* = 40)
Demographic characteristics						
Age (years)	6.26 ± 3.97	6.77 ± 4.00	4.73 ± 3.45	3.32 ± 2.31	5.63 ± 3.39	5.27 ± 4.09
Gender						
Male, *n* (%)	253 (50.20)	193 (51.06)	60 (47.62)	25 (58.14)	17 (39.53)	18 (45)
Female, *n* (%)	251 (49.80)	185 (48.94)	66 (52.38)	18 (41.86)	26 (60.47)	22 (55)
Vitamin levels						
VA (μmol/L)	0.81 ± 0.24	0.81 ± 0.26	0.81 ± 0.17	0.80 ± 0.14	0.81 ± 0.19	0.82 ± 0.19
VB1 (nmol/L)	90.43 ± 15.59	90.27 ± 15.92	90.90 ± 14.61	89.58 ± 15.19	92.27 ± 15.34	90.87 ± 13.38
VB2 (μg/L)	7.86 ± 2.73	7.83 ± 2.43	7.95 ± 3.49	8.32 ± 2.86	7.07 ± 2.79	8.49 ± 4.52
VB6 (nmol/L)	38.88 ± 10.01	40.43 ± 9.29	34.19 ± 10.64	36.11 ± 10.58	31.48 ± 10.30	34.97 ± 10.72
VB9 (nmol/L)	19.60 ± 5.01	20.33 ± 4.23	17.40 ± 6.36	17.09 ± 6.92	17.48 ± 6.30	17.66 ± 5.94
VB12 (ng/L)	435.82 ± 102.39	436.87 ± 95.83	432.62 ± 120.58	435.41 ± 108.99	424.09 ± 126.61	438.35 ± 128.47
VC (μmol/L)	41.04 ± 6.68	41.43 ± 6.99	39.87 ± 5.50	38.49 ± 3.71	40.80 ± 5.42	40.34 ± 6.88
VD (nmol/L)	61.46 ± 19.76	61.85 ± 17.45	60.26 ± 25.66	59.83 ± 26.97	57.59 ± 19.35	63.44 ± 29.87
VE (μg/mL)	10.73 ± 0.45	10.75 ± 0.43	10.65 ± 0.50	10.62 ± 0.36	10.72 ± 0.69	10.62 ± 0.37
Vitamin insufficiency rates						
VA, *n* (%)	441 (87.50)	326 (86.24)	115 (91.27)	41 (95.35)	39 (90.70)	35 (87.50)
VB1, *n* (%)	47 (9.33)	35 (9.26)	12 (9.52)	4 (9.30)	4 (9.30)	4 (10.00)
VB2, *n* (%)	4 (0.79)	0 (0.00)	4 (3.17)	1 (2.33)	0 (0.00)	3 (7.50)
VB6, *n* (%)	1 (0.20)	0 (0.00)	1 (0.79)	0 (0.00)	1 (2.33)	0 (0.00)
VB9, *n* (%)	23 (4.56)	1 (0.26)	22 (17.46)	8 (18.60)	7 (16.28)	7 (17.50)
VB12, *n* (%)	0 (0.00)	0 (0.00)	0 (0.00)	0 (0.00)	0 (0.00)	0 (0.00)
VC, *n* (%)	0 (0.00)	0 (0.00)	0 (0.00)	0 (0.00)	0 (0.00)	0 (0.00)
VD, *n* (%)	154 (30.56)	107 (28.31)	47 (37.30)	14 (32.56)	16 (37.21)	17 (42.50)
VE, *n* (%)	0 (0.00)	0 (0.00)	0 (0.00)	0 (0.00)	0 (0.00)	0 (0.00)

Abbreviations: VA, vitamin A; VB1, vitamin B1; VB2, vitamin B2; VB6, vitamin B6; VB9, vitamin B9; VB12, vitamin B12; VC, vitamin C; VD, vitamin D; VE, vitamin E.

**Table 2:** Comparison of demographic characteristics, serum vitamin levels, and vitamin insufficiency rates among the study groups.

Variables	Overall, *p*	Dis vs. Ctrl, *p*	Ecz vs. Ctrl, *p*	AD vs. Ctrl, *p*	Urt vs. Ctrl, *p*	Ecz vs. AD, *p*	Ecz vs. Urt, *p*	AD vs. Urt, *p*
Demographic characteristics								
Age (years)	<0.001 ***	<0.001 ***	<0.001 ***	0.077	0.011 *	0.001 **	0.03 *	0.355
Gender	0.310	0.504	0.379	0.152	0.466	0.084	0.231	0.614
Vitamin levels								
VA (μmol/L)	0.388	0.084	0.281	0.342	0.209	0.745	0.895	0.993
VB1 (nmol/L)	0.752	0.469	0.891	0.326	0.590	0.419	0.683	0.661
VB2 (μg/L)	0.037 *	0.279	0.269	0.008 **	0.590	0.011 *	0.353	0.241
VB6 (nmol/L)	<0.001 ***	<0.001 ***	0.003 **	<0.001 ***	0.002 **	0.044 *	0.630	0.136
VB9 (nmol/L)	<0.001 ***	<0.001 ***	<0.001 ***	0.001 **	0.005 **	0.648	0.439	0.791
VB12 (ng/L)	0.994	0.925	0.886	0.896	0.869	0.954	0.910	0.674
VC (μmol/L)	0.005 **	0.002 **	0.001 **	0.456	0.072	0.048 *	0.399	0.369
VD (nmol/L)	0.105	0.02 *	0.043 *	0.109	0.446	0.736	0.643	0.667
VE (μg/mL)	0.032 *	0.003 **	0.080	0.027 *	0.110	0.888	0.910	0.749
Vitamin insufficiency rates								
VA, *n* (%)	0.338	0.140	0.091	0.415	0.826	0.676	0.254	0.732
VB1, *n* (%)	0.990	0.910	>0.999	>0.999	0.779	>0.999	>0.999	>0.999
VB2, *n* (%)	<0.001 ***	0.004 **	0.102	—	0.001 **	>0.999	0.348	0.112
VB6, *n* (%)	0.163	0.249	—	0.100	—	0.494	—	>0.999
VB9, *n* (%)	<0.001 ***	<0.001 ***	<0.001 ***	<0.001 ***	<0.001 ***	0.591	0.896	0.690
VB12, *n* (%)	—	—	—	—	—	—	—	—
VC, *n* (%)	—	—	—	—	—	—	—	—
VD, *n* (%)	0.164	0.039 *	0.495	0.152	0.062	0.589	0.392	0.750
VE, *n* (%)	—	—	—	—	—	—	—	—

Notes: Overall *p* value were performed across four independent groups (control, eczema, atopic dermatitis, and urticaria). * *p* < 0.05, ** *p* < 0.01, *** *p* < 0.001. FDR-adjusted results for the group comparisons are summarized in Supplementary Table S2. “—” indicates that no statistical test was performed because the number of events was zero in one or more groups, or the comparison was not applicable. Abbreviations: Dis, disease group; Ctrl, control group; Ecz, eczema group; AD, atopic dermatitis group; Urt, urticaria group; VA, vitamin A; VB1, vitamin B1; VB2, vitamin B2; VB6, vitamin B6; VB9, vitamin B9; VB12, vitamin B12; VC, vitamin C; VD, vitamin D; VE, vitamin E.

**Table 3:** Univariable logistic regression analysis of demographic characteristics and vitamin-related indicators in pairwise comparisons among the study groups.

Variables	Dis vs. Ctrl, *p*	Ecz vs. Ctrl, *p*	AD vs. Ctrl, *p*	Urt vs. Ctrl, *p*	Ecz vs. AD, *p*	Ecz vs. Urt, *p*	AD vs. Urt, *p*
Gender	0.504	0.380	0.155	0.467	0.086	0.233	0.615
Age	<0.001 ***	<0.001 ***	0.075	0.027 *	0.001 **	0.014 *	0.662
VA level	0.913	0.819	0.951	0.711	0.741	0.489	0.751
VB1 level	0.695	0.787	0.437	0.817	0.414	0.679	0.657
VB2 level	0.647	0.215	0.061	0.141	0.050	0.837	0.097
VB6 level	<0.001 ***	0.005 **	<0.001 ***	0.001 **	0.049 *	0.625	0.137
VB9 level	<0.001 ***	<0.001 ***	<0.001 ***	<0.001 ***	0.786	0.685	0.890
VB12 level	0.688	0.926	0.433	0.928	0.657	0.909	0.611
VC level	0.022 *	0.002 **	0.568	0.342	0.031 *	0.141	0.729
VD level	0.438	0.504	0.143	0.613	0.662	0.563	0.298
VE level	0.03 *	0.045 *	0.630	0.070	0.394	0.909	0.451
VA insufficiency rate	0.143	0.109	0.418	0.826	0.406	0.216	0.641
VB1 insufficiency rate	0.910	0.993	0.955	0.878	0.972	0.914	0.942
VB2 insufficiency rate	0.002 **	0.030 *	—	<0.001 ***	0.474	0.298	0.095
VB6 insufficiency rate	0.137	—	0.029 *	—	0.455	—	0.485
VB9 insufficiency rate	<0.001 ***	<0.001 ***	<0.001 ***	<0.001 ***	0.592	0.896	0.691
VB12 insufficiency rate	—	—	—	—	—	—	—
VC insufficiency rate	—	—	—	—	—	—	—
VD insufficiency rate	0.039 *	0.496	0.155	0.065	0.590	0.393	0.750
VE insufficiency rate	—	—	—	—	—	—	—

Notes: This table presents only the *p* values from univariable logistic regression analyses; the corresponding ORs and 95% CIs are provided in Supplementary Table S1. * *p* < 0.05, ** *p* < 0.01, *** *p* < 0.001. FDR-adjusted results for the group comparisons are summarized in Supplementary Table S3. “—” indicates that the variable was not included in the analysis, the comparison was not applicable, or results were not reported because stable model estimation could not be achieved. Abbreviations: Dis, disease group; Ctrl, control group; Ecz, eczema group; AD, atopic dermatitis group; Urt, urticaria group; VA, vitamin A; VB1, vitamin B1; VB2, vitamin B2; VB6, vitamin B6; VB9, vitamin B9; VB12, vitamin B12; VC, vitamin C; VD, vitamin D; VE, vitamin E.

**Table 4:** Multivariable logistic regression analysis of demographic characteristics and vitamin-related indicators in pairwise comparisons among the study groups.

Variables	Dis vs. Ctrl, *p*	Ecz vs. Ctrl, *p*	AD vs. Ctrl, *p*	Urt vs. Ctrl, *p*	Ecz vs. AD, *p*	Ecz vs. Urt, *p*	AD vs. Urt, *p*
Gender	—	—	0.179	—	0.059	—	—
Age	<0.001 ***	<0.001 ***	0.079	0.046 *	0.010 *	0.035 *	—
VA level	—	—	—	—	—	—	—
VB1 level	—	—	—	—	—	—	—
VB2 level	—	—	0.036 *	0.153	0.017 *	—	0.076
VB6 level	<0.001 ***	0.02 *	<0.001 ***	0.002 **	0.029 *	—	0.104
VB9 level	<0.001 ***	0.002 **	<0.001 ***	0.001 **	—	—	—
VB12 level	—	—	—	—	—	—	—
VC level	0.072	0.046 *	—	—	0.127	0.545	—
VD level	—	—	0.941	—	—	—	—
VE level	0.216	0.206	—	0.164	—	—	—
Gender	—	—	0.188	—	0.234	—	—
Age	<0.001 ***	<0.001 ***	0.199	0.047 *	0.001 **	0.009 **	—
VA insufficiency rate	0.842	0.595	—	—	—	—	—
VB1 insufficiency rate	—	—	—	—	—	—	—
VB2 insufficiency rate	—	—	—	—	—	—	—
VB6 insufficiency rate	—	—	—	—	—	—	—
VB9 insufficiency rate	<0.001 ***	<0.001 ***	<0.001 ***	<0.001 ***	—	—	—
VB12 insufficiency rate	—	—	—	—	—	—	—
VC insufficiency rate	—	—	—	—	—	—	—
VD insufficiency rate	0.053	—	0.332	0.032 *	—	—	—
VE insufficiency rate	—	—	—	—	—	—	—

Notes: This table presents only the *p* values from multivariable logistic regression analyses; the corresponding ORs and 95% CIs are provided in Supplementary Table S2. * *p* < 0.05, ** *p* < 0.01, *** *p* < 0.001. FDR-adjusted results for the group comparisons are summarized in Supplementary Table S4. “—” indicates that the variable was not included in the analysis, the comparison was not applicable, or results were not reported because stable model estimation could not be achieved. Abbreviations: Dis, disease group; Ctrl, control group; Ecz, eczema group; AD, atopic dermatitis group; Urt, urticaria group; VA, vitamin A; VB1, vitamin B1; VB2, vitamin B2; VB6, vitamin B6; VB9, vitamin B9; VB12, vitamin B12; VC, vitamin C; VD, vitamin D; VE, vitamin E.

## Data Availability

The original contributions presented in this study are included in the article/Supplementary Materials. Further inquiries can be directed to the corresponding author.

## Supplementary Materials

The following supporting information can be downloaded at: https://www.mdpi.com/article/10.3390/nu18111754/s1, Table S1: Cut-off values used to define deficiency, insufficiency, and sufficiency for each measured vitamin; Table S2: Pairwise comparisons of demographic characteristics, serum vitamin levels, and vitamin insufficiency rates among the study groups with raw and FDR-adjusted *p* values; Table S3: Univariable logistic regression analysis of demographic characteristics and vitamin-related indicators in pairwise comparisons among the study groups; Table S4: Multivariable logistic regression analysis of demographic characteristics and vitamin-related indicators in pairwise comparisons among the study groups; Table S5: Covariate balance after matching for eczema, atopic dermatitis, and urticaria versus controls; Table S6: Univariable conditional logistic regression analyses of vitamin levels and vitamin insufficiency rates in eczema, atopic dermatitis, and urticaria versus matched controls; Table S7: Multivariable conditional logistic regression and Firth penalized regression analyses of vitamin levels and vitamin insufficiency rates in eczema, atopic dermatitis, and urticaria versus matched controls.
