# Supplementary material for: Serum Vitamin Profiles in Pediatric Eczema, Atopic Dermatitis, and Urticaria
Source: Nutrients. 2026 May 29;18(11):1754. doi: 10.3390/nu18111754 (PMC13258407; doi:10.3390/nu18111754)
Supplement: Supplementary file 1 [file nutrients-18-01754-s001.zip › nutrients-4293903-supplementary.pdf]

## Supplementary Materials

Supplementary Table S1. Cut-off values used to define deficiency, insufficiency, and sufficiency for each measured vitamin.

| Vitamins | Deficiency | Insufficiency | Sufficiency |
|---|---|---|---|
| VA (μmol/L) | <0.7 | 0.7-1.05 | >1.05-1.75 |
| VB1 (nmol/L) | <70 | - | 70-180 |
| VB2 (μg/L) | <4.26 | - | 4.26-18.42 |
| VB6 (nmol/L) | <14.6 | - | 14.6-72.9 |
| VB9 (nmol/L) | <6.8 | 6.8-11.3 | >11.3-36.3 |
| VB12 (ng/L) | <100 | 100-200 | >200-900 |
| VC (μmol/L) | <11.4 | 11.4-22.7 | >22.7 |
| VD (nmol/L) | <37.5 | 37.5-50 | >50-250 |
| VE (μg/mL) | <5 | 5-7 | >7-20 |

Note: Vitamin status was classified according to national pediatric guidelines and published references[1,2]. A dash (–) indicates that no insufficiency category was defined for that vitamin. In the statistical analyses, deficiency and insufficiency were combined and analyzed as vitamin insufficiency. Abbreviations: VA, vitamin A; VB1, vitamin B1; VB2, vitamin B2; VB6, vitamin B6; VB9, vitamin B9 (folate); VB12, vitamin B12; VC, vitamin C; VD, vitamin D; VE, vitamin E.

Supplementary Table S2. Pairwise comparisons of demographic characteristics, serum vitamin levels, and vitamin insufficiency rates among the study groups with raw and FDR-adjusted P values.

| **Variables** | **Overall** | **Dis vs. Ctrl** | | **Ecz vs. Ctrl** | | **AD vs. Ctrl** | | **Urt vs. Ctrl** | | **Ecz vs. AD** | | **Ecz vs. Urt** | | **AD vs. Urt** | |
|---|---|---|---|---|---|---|---|---|---|---|---|---|---|---|---|
| | **Raw P** | **Raw P** | **FDR-adjusted P** | **Raw P** | **FDR-adjusted P** | **Raw P** | **FDR-adjusted P** | **Raw P** | **FDR-adjusted P** | **Raw P** | **FDR-adjusted P** | **Raw P** | **FDR-adjusted P** | **Raw P** | **FDR-adjusted P** |
| Demographic characteristics | | | | | | | | | | | | | | | |
| Age (years) | <0.001*** | <0.001*** | <0.001*** | <0.001*** | <0.001*** | 0.077 | 0.268 | 0.011* | 0.057† | 0.001** | 0.007** | 0.03* | 0.132† | 0.355 | 0.691 |
| Gender | 0.310 | 0.504 | 0.794 | 0.379 | 0.714 | 0.152 | 0.381 | 0.466 | 0.771 | 0.084 | 0.27 | 0.231 | 0.554 | 0.614 | 0.906 |
| Vitamin levels | | | | | | | | | | | | | | | |
| VA (μmol/L) | 0.388 | 0.084 | 0.270 | 0.281 | 0.599 | 0.342 | 0.691 | 0.209 | 0.512 | 0.745 | 0.938 | 0.895 | 0.997 | 0.993 | >0.999 |
| VB1 (nmol/L) | 0.752 | 0.469 | 0.771 | 0.891 | 0.997 | 0.326 | 0.682 | 0.590 | 0.883 | 0.419 | 0.741 | 0.683 | 0.913 | 0.661 | 0.913 |
| VB2 (μg/L) | 0.037* | 0.279 | 0.599 | 0.269 | 0.595 | 0.008** | 0.046* | 0.590 | 0.883 | 0.011* | 0.057† | 0.353 | 0.691 | 0.241 | 0.565 |
| VB6 (nmol/L) | <0.001*** | <0.001*** | <0.001*** | 0.003** | 0.021* | <0.001*** | <0.001*** | 0.002** | 0.014* | 0.044* | 0.175† | 0.630 | 0.913 | 0.136 | 0.363 |
| VB9 (nmol/L) | <0.001*** | <0.001*** | <0.001*** | <0.001*** | 0.003** | 0.001** | 0.012* | 0.005** | 0.030* | 0.648 | 0.913 | 0.439 | 0.764 | 0.791 | 0.968 |
| VB12 (ng/L) | 0.994 | 0.925 | >0.999 | 0.886 | 0.997 | 0.896 | 0.997 | 0.869 | 0.997 | 0.954 | >0.999 | 0.910 | 0.997 | 0.674 | 0.913 |
| VC (μmol/L) | 0.005** | 0.002** | 0.016* | 0.001** | 0.010* | 0.456 | 0.771 | 0.072 | 0.258 | 0.048* | 0.186† | 0.399 | 0.729 | 0.369 | 0.708 |
| VD (nmol/L) | 0.105 | 0.020* | 0.095† | 0.043* | 0.175† | 0.109 | 0.305 | 0.446 | 0.765 | 0.736 | 0.938 | 0.643 | 0.913 | 0.667 | 0.913 |
| VE (μg/mL) | 0.032* | 0.003** | 0.021* | 0.080 | 0.27 | 0.027* | 0.123† | 0.110 | 0.305 | 0.888 | 0.997 | 0.910 | 0.997 | 0.749 | 0.938 |
| Vitamin insufficiency rates | | | | | | | | | | | | | | | |
| VA, n (%) | 0.338 | 0.140 | 0.365 | 0.091 | 0.282 | 0.415 | 0.741 | 0.826 | 0.997 | 0.676 | 0.913 | 0.254 | 0.572 | 0.732 | 0.938 |
| VB1, n (%) | 0.990 | 0.910 | 0.997 | >0.999 | 1.000 | >0.999 | >0.999 | 0.779 | 0.963 | >0.999 | >0.999 | >0.999 | >0.999 | >0.999 | >0.999 |
| VB2, n (%) | <0.001*** | 0.004** | 0.022* | 0.102 | 0.301 | — | — | 0.001** | 0.008** | >0.999 | >0.999 | 0.348 | 0.691 | 0.112 | 0.305 |
| VB6, n (%) | 0.163 | 0.249 | 0.572 | — | — | 0.100 | 0.301 | — | — | 0.494 | 0.791 | — | — | >0.999 | >0.999 |
| VB9, n (%) | <0.001*** | <0.001*** | <0.001*** | <0.001*** | <0.001*** | <0.001*** | <0.001*** | <0.001*** | <0.001*** | 0.591 | 0.883 | 0.896 | 0.997 | 0.690 | 0.913 |
| VB12, n (%) | — | — | — | — | — | — | — | — | — | — | — | — | — | — | — |
| VC, n (%) | — | — | — | — | — | — | — | — | — | — | — | — | — | — | — |
| VD, n (%) | 0.164 | 0.039* | 0.165† | 0.495 | 0.791 | 0.152 | 0.381 | 0.062 | 0.229 | 0.589 | 0.883 | 0.392 | 0.727 | 0.750 | 0.938 |
| VE, n (%) | — | — | — | — | — | — | — | — | — | — | — | — | — | — | — |

Notes: Overall P value were performed across four independent groups (control, eczema, atopic dermatitis, and urticaria). Raw P values and FDR-adjusted P values are shown. *P < 0.05, **P < 0.01, and ***P < 0.001. † Indicates results that were significant in the raw analysis but no longer significant after FDR correction. "—" indicates that no statistical test was performed because the number of events was zero in one or more groups, or the comparison was not applicable.

Abbreviations: Dis, disease group; Ctrl, control group; Ecz, eczema group; AD, atopic dermatitis group; Urt, urticaria group; VA, vitamin A; VB1, vitamin B1; VB2, vitamin B2; VB6, vitamin B6; VB9, vitamin B9; VB12, vitamin B12; VC, vitamin C; VD, vitamin D; VE, vitamin E.

Supplementary Table S3. Univariable logistic regression analysis of demographic characteristics and vitamin-related indicators in pairwise comparisons among the study groups

| **Variables** | **Dis vs. Ctrl** | | | **Ecz vs. Ctrl** | | | **AD vs. Ctrl** | | | **Urt vs. Ctrl** | | | **Ecz vs. AD** | | | **Ecz vs. Urt** | | | **AD vs. Urt** | | |
|---|---|---|---|---|---|---|---|---|---|---|---|---|---|---|---|---|---|---|---|---|---|
| | **OR (95% CI)** | **RAW P** | **FDR P** | **OR (95% CI)** | **RAW P** | **FDR P** | **OR (95% CI)** | **RAW P** | **FDR P** | **OR (95% CI)** | **RAW P** | **FDR P** | **OR (95% CI)** | **RAW P** | **FDR P** | **OR (95% CI)** | **RAW P** | **FDR P** | **OR (95% CI)** | **RAW P** | **FDR P** |
| Gender | 0.87 (0.58–1.30) | 0.504 | 0.805 | 1.33 (0.70–2.52) | 0.380 | 0.768 | 0.63 (0.33–1.19) | 0.155 | 0.371 | 0.78 (0.41–1.51) | 0.467 | 0.802 | 2.12 (0.90–5.02) | 0.086 | 0.253 | 1.70 (0.71–4.05) | 0.233 | 0.452 | 0.80 (0.33–1.91) | 0.615 | 0.781 |
| Age | 0.86 (0.81–0.91) | <0.001*** | <0.001*** | 0.72 (0.63–0.82) | <0.001*** | <0.001*** | 0.92 (0.85–1.01) | 0.075 | 0.235 | 0.90 (0.82–0.99) | 0.027* | 0.124† | 0.75 (0.63–0.89) | 0.001** | 0.008** | 0.82 (0.71–0.96) | 0.014* | 0.075† | 1.03 (0.91–1.15) | 0.662 | 0.843 |
| VA level | 1.05 (0.45–2.43) | 0.913 | 0.939 | 0.86 (0.23–3.18) | 0.819 | 0.898 | 1.04 (0.30–3.62) | 0.951 | 0.951 | 1.26 (0.37–4.37) | 0.711 | 0.851 | 0.65 (0.05–8.68) | 0.741 | 0.865 | 0.39 (0.03–5.70) | 0.489 | 0.805 | 0.69 (0.07–6.91) | 0.751 | 0.865 |
| VB1 level | 1.00 (0.99–1.02) | 0.695 | 0.843 | 1.00 (0.98–1.02) | 0.787 | 0.885 | 1.01 (0.99–1.03) | 0.437 | 0.798 | 1.00 (0.98–1.02) | 0.817 | 0.898 | 0.99 (0.96–1.02) | 0.414 | 0.798 | 0.99 (0.96–1.02) | 0.679 | 0.843 | 1.01 (0.98–1.04) | 0.657 | 0.843 |
| VB2 level | 1.02 (0.95–1.09) | 0.647 | 0.843 | 1.08 (0.96–1.21) | 0.215 | 0.501 | 0.86 (0.74–1.01) | 0.061 | 0.214 | 1.08 (0.97–1.21) | 0.141 | 0.362 | 1.18 (1.00–1.39) | 0.050 | 0.183 | 0.99 (0.88–1.11) | 0.837 | 0.907 | 0.90 (0.79–1.02) | 0.097 | 0.276 |
| VB6 level | 0.93 (0.91–0.95) | <0.001*** | <0.001*** | 0.95 (0.91–0.98) | 0.005** | 0.030* | 0.89 (0.85–0.93) | <0.001*** | <0.001*** | 0.93 (0.90–0.97) | 0.001** | 0.006** | 1.04 (1.00–1.09) | 0.049* | 0.183† | 1.01 (0.97–1.05) | 0.625 | 0.843 | 0.97 (0.93–1.01) | 0.137 | 0.362 |
| VB9 level | 0.88 (0.84–0.92) | <0.001*** | <0.001*** | 0.84 (0.78–0.91) | <0.001*** | <0.001*** | 0.86 (0.79–0.93) | <0.001*** | 0.002** | 0.86 (0.79–0.94) | <0.001*** | 0.004** | 0.99 (0.93–1.06) | 0.786 | 0.885 | 0.99 (0.92–1.05) | 0.685 | 0.843 | 0.99 (0.93–1.07) | 0.890 | 0.939 |
| VB12 level | 1.00 (1.00–1.00) | 0.688 | 0.843 | 1.00 (1.00–1.00) | 0.926 | 0.939 | 1.00 (1.00–1.00) | 0.433 | 0.798 | 1.00 (1.00–1.00) | 0.928 | 0.939 | 1.00 (1.00–1.00) | 0.657 | 0.843 | 1.00 (1.00–1.00) | 0.909 | 0.939 | 1.00 (1.00–1.00) | 0.611 | 0.843 |
| VC level | 0.96 (0.92–0.99) | 0.022* | 0.111† | 0.89 (0.82–0.96) | 0.002** | 0.016* | 0.98 (0.93–1.04) | 0.568 | 0.843 | 0.97 (0.92–1.03) | 0.342 | 0.723 | 0.89 (0.80–0.99) | 0.031* | 0.126† | 0.93 (0.85–1.02) | 0.141 | 0.362 | 1.01 (0.94–1.09) | 0.729 | 0.861 |
| VD level | 1.00 (0.99–1.01) | 0.438 | 0.798 | 0.99 (0.98–1.01) | 0.504 | 0.805 | 0.99 (0.97–1.00) | 0.143 | 0.362 | 1.00 (0.99–1.02) | 0.613 | 0.843 | 1.00 (0.99–1.02) | 0.662 | 0.843 | 1.00 (0.98–1.01) | 0.563 | 0.843 | 0.99 (0.97–1.01) | 0.298 | 0.647 |
| VE level | 0.57 (0.34–0.95) | 0.03* | 0.126† | 0.40 (0.16–0.98) | 0.045* | 0.177† | 0.84 (0.40–1.73) | 0.630 | 0.843 | 0.43 (0.17–1.07) | 0.070 | 0.235 | 0.70 (0.31–1.59) | 0.394 | 0.779 | 0.93 (0.28–3.06) | 0.909 | 0.939 | 1.37 (0.60–3.11) | 0.451 | 0.802 |
| VA insufficiency rate | 1.67 (0.84–3.31) | 0.143 | 0.33 | 3.27 (0.77–13.92) | 0.109 | 0.277 | 1.56 (0.53–4.53) | 0.418 | 0.690 | 1.12 (0.42–2.98) | 0.826 | 0.940 | 2.10 (0.36–12.14) | 0.406 | 0.687 | 2.93 (0.54–16.04) | 0.216 | 0.446 | 1.39 (0.35–5.60) | 0.641 | 0.794 |
| VB1 insufficiency rate | 1.04 (0.52–2.07) | 0.910 | 0.973 | 1.01 (0.34–2.98) | 0.993 | 0.993 | 1.03 (0.35–3.06) | 0.955 | 0.985 | 1.09 (0.37–3.24) | 0.878 | 0.973 | 0.97 (0.23–4.18) | 0.972 | 0.987 | 0.92 (0.22–3.97) | 0.914 | 0.973 | 0.95 (0.22–4.08) | 0.942 | 0.985 |
| VB2 insufficiency rate | 28.03 (2.96–3728.62) | 0.002** | 0.010* | 26.73 (1.40–3926.50) | 0.030* | 0.111† | — | — | — | 70.65 (6.67–9584.50) | <0.001*** | 0.001** | 3.00 (0.16–443.20) | 0.474 | 0.693 | 0.29 (0.03–2.95) | 0.298 | 0.562 | 0.126 (0.001–1.362) | 0.095 | 0.249 |
| VB6 insufficiency rate | 9.12 (0.48–1333.60) | 0.137 | 0.330 | — | — | — | 27.36 (1.44–4016.80) | 0.029* | 0.111† | — | — | — | 0.32 (0.002–6.13) | 0.455 | 0.693 | — | — | — | 2.93 (0.152–432.8) | 0.485 | 0.693 |
| VB9 insufficiency rate | 76.13 (10.12–572.59) | <0.001*** | <0.001*** | 86.17 (10.47–709.02) | <0.001*** | <0.001*** | 62.83 (7.36–536.44) | <0.001*** | 0.001** | 79.97 (9.55–669.80) | <0.001*** | <0.001*** | 1.37 (0.43–4.36) | 0.592 | 0.781 | 1.08 (0.35–3.30) | 0.896 | 0.973 | 0.79 (0.24–2.58) | 0.691 | 0.814 |
| VB12 insufficiency rate | — | — | — | — | — | — | — | — | — | — | — | — | — | — | — | — | — | — | — | — | — |
| VC insufficiency rate | — | — | — | — | — | — | — | — | — | — | — | — | — | — | — | — | — | — | — | — | — |
| VD insufficiency rate | 1.57 (1.02–2.40) | 0.039* | 0.135† | 1.27 (0.64–2.50) | 0.496 | 0.693 | 1.62 (0.83–3.16) | 0.155 | 0.330 | 1.87 (0.96–3.64) | 0.065 | 0.215 | 0.78 (0.32–1.92) | 0.590 | 0.781 | 0.68 (0.28–1.66) | 0.393 | 0.683 | 0.87 (0.36–2.10) | 0.75 | 0.868 |
| VE insufficiency rate | — | — | — | — | — | — | — | — | — | — | — | — | — | — | — | — | — | — | — | — | — |

Note: Odds ratios (ORs) with 95% confidence intervals (CIs), raw P values, and false discovery rate (FDR)-adjusted P values are presented. *P < 0.05, **P < 0.01, ***P < 0.001. † Indicates results that were significant in the raw analysis but no longer significant after FDR correction. "—" indicates that the variable was not included in the analysis, the comparison was not applicable, or results were not reported because stable model estimation could not be achieved. Abbreviations: Dis, disease group; Ctrl, control group; Ecz, eczema group; AD, atopic dermatitis group; Urt, urticaria group; OR, odds ratio; CI, confidence interval; VA, vitamin A; VB1, vitamin B1; VB2, vitamin B2; VB6, vitamin B6; VB9, vitamin B9; VB12, vitamin B12; VC, vitamin C; VD, vitamin D; VE, vitamin E.

Supplementary Table S4. Multivariable logistic regression analysis of demographic characteristics and vitamin-related indicators in pairwise comparisons among the study groups

| Variables | Dis vs. Ctrl | | | Ecz vs. Ctrl | | | AD vs. Ctrl | | | Urt vs. Ctrl | | | Ecz vs. AD | | | Ecz vs. Urt | | | AD vs. Urt | | |
|---|---|---|---|---|---|---|---|---|---|---|---|---|---|---|---|---|---|---|---|---|---|
| | OR (95% CI) | RAW P | FDR P | OR (95% CI) | RAW P | FDR P | OR (95% CI) | RAW P | FDR P | OR (95% CI) | RAW P | FDR P | OR (95% CI) | RAW P | FDR P | OR (95% CI) | RAW P | FDR P | OR (95% CI) | RAW P | FDR P |
| Gender | — | — | — | — | — | — | 0.60 (0.29–1.26) | 0.179 | 0.206 | — | — | — | 2.80 (0.96–8.14) | 0.059 | 0.098 | — | — | — | — | — | — |
| Age | 0.87 (0.82–0.93) | <0.001*** | <0.001*** | 0.74 (0.64–0.85) | <0.001*** | <0.001*** | 0.91 (0.82–1.01) | 0.079 | 0.112 | 0.91 (0.82–1.00) | 0.046* | 0.082† | 0.78 (0.65–0.94) | 0.010* | 0.029* | 0.84 (0.71–0.99) | 0.035* | 0.072† | — | — | — |
| VA level | — | — | — | — | — | — | — | — | — | — | — | — | — | — | — | — | — | — | — | — | — |
| VB1 level | — | — | — | — | — | — | — | — | — | — | — | — | — | — | — | — | — | — | — | — | — |
| VB2 level | — | — | — | — | — | — | 0.82 (0.68–0.99) | 0.036* | 0.072† | 1.09 (0.97–1.22) | 0.153 | 0.192 | 1.29 (1.05–1.59) | 0.017* | 0.047* | — | — | — | 0.89 (0.78–1.01) | 0.076 | 0.112 |
| VB6 level | 0.93 (0.90–0.95) | <0.001*** | <0.001*** | 0.95 (0.91–0.99) | 0.02* | 0.049* | 0.88 (0.84–0.92) | <0.001*** | <0.001*** | 0.94 (0.90–0.98) | 0.002** | 0.006** | 1.06 (1.01–1.11) | 0.029* | 0.068† | — | — | — | 0.96 (0.92–1.01) | 0.104 | 0.142 |
| VB9 level | 0.89 (0.85–0.94) | <0.001*** | <0.001*** | 0.89 (0.82–0.96) | 0.002** | 0.006** | 0.86 (0.79–0.93) | <0.001*** | 0.002** | 0.87 (0.80–0.95) | 0.001** | 0.005** | — | — | — | — | — | — | — | — | — |
| VB12 level | — | — | — | — | — | — | — | — | — | — | — | — | — | — | — | — | — | — | — | — | — |
| VC level | 0.96 (0.92–1.00) | 0.072 | 0.112 | 0.92 (0.85–1.00) | 0.046* | 0.082† | — | — | — | — | — | — | 0.90 (0.79–1.03) | 0.127 | 0.166 | 0.97 (0.88–1.07) | 0.545 | 0.564 | — | — | — |
| VD level | — | — | — | — | — | — | 1.00 (0.98–1.03) | 0.941 | 0.941 | — | — | — | — | — | — | — | — | — | — | — | — |
| VE level | 0.72 (0.42–1.21) | 0.216 | 0.231 | 0.55 (0.22–1.39) | 0.206 | 0.229 | — | — | — | 0.52 (0.21–1.30) | 0.164 | 0.196 | — | — | — | — | — | — | — | — | — |
| Gender | — | — | — | — | — | — | 0.63 (0.31–1.25) | 0.188 | 0.260 | — | — | — | 1.73 (0.70–4.33) | 0.234 | 0.284 | — | — | — | — | — | — |
| Age | 0.88 (0.82–0.93) | <0.001*** | <0.001*** | 0.76 (0.66–0.86) | <0.001*** | <0.001*** | 0.94 (0.86–1.03) | 0.199 | 0.260 | 0.91 (0.83–1.00) | 0.047* | 0.079† | 0.77 (0.64–0.90) | 0.001** | 0.002** | 0.83 (0.71–0.96) | 0.009** | 0.018* | — | — | — |
| VA insufficiency rate | 1.08 (0.54–2.32) | 0.842 | 0.842 | 1.42 (0.43–7.28) | 0.595 | 0.633 | — | — | — | — | — | — | — | — | — | — | — | — | — | — | — |
| VB1 insufficiency rate | — | — | — | — | — | — | — | — | — | — | — | — | — | — | — | — | — | — | — | — | — |
| VB2 insufficiency rate | — | — | — | — | — | — | — | — | — | — | — | — | — | — | — | — | — | — | — | — | — |
| VB6 insufficiency rate | — | — | — | — | — | — | — | — | — | — | — | — | — | — | — | — | — | — | — | — | — |
| VB9 insufficiency rate | 45.40 (10.94–423.00) | <0.001*** | <0.001*** | 28.73 (6.00–279.50) | <0.001*** | <0.001*** | 37.06 (7.29–369.60) | <0.001*** | <0.001*** | 60.76 (11.85–626.70) | <0.001*** | <0.001*** | — | — | — | — | — | — | — | — | — |
| VB12 insufficiency rate | — | — | — | — | — | — | — | — | — | — | — | — | — | — | — | — | — | — | — | — | — |
| VC insufficiency rate | — | — | — | — | — | — | — | — | — | — | — | — | — | — | — | — | — | — | — | — | — |
| VD insufficiency rate | 1.58 (0.995–2.51) | 0.053 | 0.081 | — | — | — | 1.43 (0.69–2.86) | 0.332 | 0.376 | 2.20 (1.07–4.47) | 0.032* | 0.061† | — | — | — | — | — | — | — | — | — |
| VE insufficiency rate | — | — | — | — | — | — | — | — | — | — | — | — | — | — | — | — | — | — | — | — | — |

Note: Odds ratios (ORs) with 95% confidence intervals (CIs), raw P values, and false discovery rate (FDR)-adjusted P values are presented. *P < 0.05, **P < 0.01, ***P < 0.001. † Indicates results that were significant in the raw analysis but no longer significant after FDR correction. "—" indicates that the variable was not included in the analysis, the comparison was not applicable, or results were not reported because stable model estimation could not be achieved. Abbreviations: Dis, disease group; Ctrl, control group; Ecz, eczema group; AD, atopic dermatitis group; Urt, ur-ticaria group; OR, odds ratio; CI, confidence interval; VA, vitamin A; VB1, vitamin B1; VB2, vitamin B2; VB6, vitamin B6; VB9, vitamin B9; VB12, vitamin B12; VC, vitamin C; VD, vitamin D; VE, vitamin E.

Supplementary Table S5. Covariate balance after matching for eczema, atopic dermatitis, and urticaria versus controls

| Comparison | Covariate | Means Treated | Means Control | Std. Mean Diff. | Var. Ratio | eCDF Max |
|---|---|---|---|---|---|---|
| Ecz vs. Ctrl | distance | 0.1828 | 0.1817 | 0.011 | 1.0254 | 0.093 |
| | Age | 3.3193 | 3.4401 | -0.0523 | 0.9624 | 0.1163 |
| | Gender | 0.5814 | 0.6744 | -0.1886 | NA | 0.093 |
| AD vs. Ctrl | distance | 0.1122 | 0.1122 | -0.0007 | 0.9938 | 0.0698 |
| | Age | 5.6279 | 5.6282 | -0.0001 | 0.8388 | 0.1163 |
| | Gender | 0.3953 | 0.3953 | 0 | NA | 0 |
| Urt vs. Ctrl | distance | 0.1084 | 0.1082 | 0.0034 | 1.0044 | 0.075 |
| | Age | 5.2721 | 5.1757 | 0.0236 | 1.0543 | 0.125 |
| | Gender | 0.45 | 0.5 | -0.1005 | NA | 0.05 |

Note: Covariate balance after matching is summarized by treated and control means, standardized mean differences (SMDs), variance ratios, and eCDF maximum differences. Absolute SMDs closer to 0 indicate better balance; values < 0.1 are generally considered well balanced, 0.1–0.2 indicate acceptable balance, and > 0.2 indicate substantial imbalance. "Distance" represents the estimated propensity score distance used in the matching procedure. Abbreviations: Ecz, eczema; AD, atopic dermatitis; Urt, urticaria; Ctrl, control; eCDF, empirical cumulative distribution function.

Supplementary Table S6. Univariable conditional logistic regression analyses of vitamin levels and vitamin insufficiency rates in eczema, atopic dermatitis, and urticaria versus matched controls

| Variables | Ecz vs. Ctrl | | AD vs. Ctrl | | Urt vs. Ctrl | |
|---|---|---|---|---|---|---|
| | OR (95% CI) | P | OR (95% CI) | P | OR (95% CI) | P |
| VA level | 0.76 (0.09–6.52) | 0.798 | 0.75 (0.12–4.67) | 0.754 | 3.79 (0.34–41.76) | 0.277 |
| VB1 level | 1.00 (0.97–1.03) | 0.968 | 1.01 (0.98–1.04) | 0.456 | 1.00 (0.97–1.03) | 0.843 |
| VB2 level | 1.08 (0.92–1.27) | 0.327 | 0.89 (0.75–1.05) | 0.158 | 1.03 (0.91–1.17) | 0.613 |
| VB6 level | 0.94 (0.90–0.99) | 0.018* | 0.87 (0.80–0.95) | 0.001** | 0.93 (0.88–0.98) | 0.012* |
| VB9 level | 0.93 (0.86–1.00) | 0.047* | 0.91 (0.83–0.99) | 0.029* | 0.95 (0.88–1.03) | 0.229 |
| VB12 level | 1.00 (1.00–1.00) | 0.945 | 1.00 (1.00–1.00) | 0.756 | 1.00 (1.00–1.00) | 0.731 |
| VC level | 0.94 (0.84–1.04) | 0.237 | 1.03 (0.94–1.14) | 0.516 | 1.06 (0.97–1.16) | 0.174 |
| VD level | 0.99 (0.97–1.01) | 0.267 | 0.98 (0.95–1.00) | 0.111 | 1.01 (0.99–1.02) | 0.578 |
| VE level | 0.97 (0.29–3.21) | 0.958 | 1.49 (0.62–3.60) | 0.374 | 0.69 (0.27–1.79) | 0.449 |
| VA insufficiency rate | 2.88 (0.69–16.43) | 0.153 | 2.00 (0.60–6.64) | 0.258 | 1.45 (0.44–5.03) | 0.545 |
| VB1 insufficiency rate | 1.32 (0.30–6.24) | 0.71 | 1.94 (0.40–11.66) | 0.409 | 1.00 (0.24–4.16) | 1 |
| VB2 insufficiency rate | 3.07 (0.16–453.25) | 0.465 | — | — | 7.56 (0.70–1029.49) | 0.104 |
| VB6 insufficiency rate | — | — | 3.14 (0.16–464.24) | 0.455 | — | — |
| VB9 insufficiency rate | 20.83 (2.45–2727.83) | 0.002** | 15.49 (1.73–2045.94) | 0.01* | 18.13 (2.08–2385.59) | 0.005** |
| VB12 insufficiency rate | — | — | — | — | — | — |
| VC insufficiency rate | — | — | — | — | — | — |
| VD insufficiency rate | 2.00 (0.75–5.33) | 0.166 | 2.40 (0.85–6.81) | 0.1 | 2.20 (0.76–6.33) | 0.144 |
| VE insufficiency rate | — | — | — | — | — | — |

Note: Odds ratios (ORs) with 95% confidence intervals (CIs), and P values are presented. *P < 0.05, **P < 0.01, ***P < 0.001. "—" indicates that the variable was not included in the analysis, the comparison was not applicable, or results were not reported because stable model estimation could not be achieved. Abbreviations: Ctrl, control group; Ecz, eczema group; AD, atopic dermatitis group; Urt, urticaria group; OR, odds ratio; CI, confidence interval; VA, vitamin A; VB1, vitamin B1; VB2, vitamin B2; VB6, vitamin B6; VB9, vitamin B9; VB12, vitamin B12; VC, vitamin C; VD, vitamin D; VE, vitamin E.

Supplementary Table S7. Multivariable conditional logistic regression and Firth penalized regression analyses of vitamin levels and vitamin insufficiency rates in eczema, atopic dermatitis, and urticaria versus matched controls

| Variables | Ecz vs. Ctrl | | AD vs. Ctrl | | Urt vs. Ctrl | |
|---|---|---|---|---|---|---|
| | **OR (95% CI)** | **P** | **OR (95% CI)** | **P** | **OR (95% CI)** | **P** |
| VA level | — | — | — | — | — | — |
| VB1 level | — | — | — | — | — | — |
| VB2 level | — | — | 0.75 (0.54–1.04) | 0.084 | — | — |
| VB6 level | 0.95 (0.90–1.00) | 0.039* | 0.85 (0.76–0.95) | 0.004** | 0.94 (0.88–0.99) | 0.022* |
| VB9 level | 0.94 (0.87–1.01) | 0.112 | 0.79 (0.63–0.98) | 0.031* | — | — |
| VB12 level | — | — | — | — | — | — |
| VC level | — | — | — | — | 1.03 (0.93–1.14) | 0.532 |
| VD level | — | — | 0.96 (0.92–1.02) | 0.169 | — | — |
| VE level | — | — | — | — | — | — |
| VA insufficiency rate | 2.58(0.59–15.40) | 0.215 | — | — | — | — |

| | | | | | | |
|---|---|---|---|---|---|---|
| VB1 insufficiency rate | — | — | — | — | — | — |
| VB2 insufficiency rate | — | — | — | — | 5.97 (0.50–835.19) | 0.176 |
| VB6 insufficiency rate | — | — | — | — | — | — |
| VB9 insufficiency rate | 18.50 (2.13–2434.59) | 0.004** | 14.50 (1.60–1920.75) | 0.013* | 23.35 (2.60–3094.57) | 0.002** |
| VB12 insufficiency rate | — | — | — | — | — | — |
| VC insufficiency rate | — | — | — | — | — | — |
| VD insufficiency rate | 2.20 (0.78–6.51) | 0.135 | 1.91 (0.73–5.11) | 0.187 | 1.98 (0.74–5.38) | 0.173 |
| VE insufficiency rate | — | — | — | — | — | — |

Note: Odds ratios (ORs) with 95% confidence intervals (CIs), and P values are presented. *P < 0.05, **P < 0.01, ***P < 0.001. "—" indicates that the variable was not included in the analysis, the comparison was not applicable, or results were not reported because stable model estimation could not be achieved. Abbreviations: Ctrl, control group; Ecz, eczema group; AD, atopic dermatitis group; Urt, urticaria group; OR, odds ratio; CI, confidence interval; VA, vitamin A; VB1, vitamin B1; VB2, vitamin B2; VB6, vitamin B6; VB9, vitamin B9; VB12, vitamin B12; VC, vitamin C; VD, vitamin D; VE, vitamin E.

## References


1. Whitfield KC, Bourassa MW, Adamolekun B, et al. Thiamine deficiency disorders: diagnosis, prevalence, and a roadmap for global control programs. *Annals of the New York Academy of Sciences*. 2018;1430(1):3-43. doi:10.1111/nyas.13919

2. Subspecialty Group of Child Health Care, Society of Pediatrics, Chinese Medical Association, Editorial Board of Chinese Journal of Pediatrics. [Recommendations for prevention and treatment of trace nutrients deficiency in children]. *Zhonghua Er Ke Za Zhi*. 2010;48(7):502-509.
